# Evaluation of Placental Toxicity of Five Essential Oils and Their Potential Endocrine-Disrupting Effects

**DOI:** 10.3390/cimb44070192

**Published:** 2022-06-28

**Authors:** Sophie Fouyet, Elodie Olivier, Pascale Leproux, Mélody Dutot, Patrice Rat

**Affiliations:** 1 CNRS CiTCoM, Université de Paris Cité, 75006 Paris, France; elodie.olivier@u-paris.fr (E.O.); pascale.leproux@u-paris.fr (P.L.); melody.dutot@yslab.fr (M.D.); patrice.rat@u-paris.fr (P.R.); 2 Laboratoires Léa Nature, 17180 Périgny, France; 3 Recherche & Développement, Yslab, 29000 Quimper, France

**Keywords:** essential oil, placental toxicity, endocrine disruptor, P2X7 receptor, hormones

## Abstract

Pregnant women may use EOs in case of morning sickness, nausea, stress management, etc. Little is known about the potential danger that EOs represent for the placenta and therefore for the pregnancy. Our aim was to explore and compare the placental toxicity and potential endocrine disrupting effects of niaouli, orange, tea tree, wintergreen and ylang-ylang EOs, and their key compounds: 4-terpineol, 1,8-cineol, limonene, methyl salicylate and benzyl salicylate. We studied the release of four hormones and the activation of P2X7 receptor in JEG-Tox human placental cells as key biomarkers for endocrine toxicity. We observed that niaouli, orange, tea tree, wintergreen and ylang-ylang EOs and their key components disrupted at least one of the studied hormones but none of them activated the P2X7 cell death receptor. The tested EOs appear then to be more hormonal modulators rather than EDCs in human placental cells. The hormonal effects observed with the key components were very different from those observed with the EOs. EOs are very complex mixtures, and it is essential to study whole EOs rather than their components individually in safety assessment.

## 1. Introduction

Currently, there is a trend in developed countries towards a more natural lifestyle with less attraction to medical treatments and an increasing use of natural products such as essential oils (EOs). Empirical data say that EOs have many beneficial properties for health, for example niaouli and tea tree EOs possess antiseptic, antiviral and antifungal properties; orange and ylang-ylang EOs decrease stress and anxiety; and wintergreen EO reduces inflammation. EOs are also used in a multitude of daily life products including food flavorings, soaps, lotions, shampoos, hair styling products, cologne, laundry detergents and even insect repellents [1]. Routes of exposure to EOs are consequently diverse: inhalation, ingestion, and contact with skin.

Pregnancy is a time when various complications can occur. However, it is also a time when many drugs are contraindicated. That is why many pregnant women prefer to use herbs, herbal preparations and EOs to treat pregnancy-related symptoms and minor disorders. EOs are then used in different situations: in diffusion devices to purify the atmosphere of the house, decrease anxiety and stress, and improve the quality of sleep; in inhalers to treat respiratory infections such as cold, sinusitis and bronchitis; and in skin preparations to reduce neuralgia, digestive disorders, pain and to prevent or treat stretch marks or even to prepare childbirth. They may also use EOs in commercially available or homemade household products for cleaning to avoid chemicals contained in traditional cleansing products that they perceive as hazardous for their pregnancy.

Many people, including pregnant women, seem to consider EOs as safe alternatives because of their natural origin, but natural does not mean safe. Women often use aromatherapy without medical advice during pregnancy [2] and randomly search on the web to find information that is most of the time unreliable. EOs can induce side effects such as irritation, photosensitization and even abortion [3]. In this context, it is of most importance to highlight that the use of EOs requires knowledge, especially during pregnancy since pregnancy is a time of great vulnerability. EOs, composed of small and liposoluble molecules, can pass into the blood circulation after diffusion through skin, pulmonary and digestive barriers, ultimately reaching the placenta where they can accumulate [4] and even cross the placental barrier [5]. The placenta is a unique, transient organ shared by two organisms [6]. It supports the normal growth and development of the fetus by coordinating exchanges of nutrients and wastes between maternal and fetal circulatory systems, and it also allows a bidirectional hormonal regulation of the mother and her fetus [6]. Therefore, any toxic molecule that pregnant women are exposed to may induce placental dysfunctions, leading to pregnancy disorders, such as miscarriage or preeclampsia [7,8,9,10], and can have short- and long-term consequences for both the mother and the unborn child [7,11].

Some studies have reported cases of prepubertal gynecomastia and premature thelarche in children after the use of lavender and tea tree EOs [12,13], suggesting that lavender EO, tea tree EO and some of their compounds (linalool, α-terpineol and 4-terpinenol) have endocrine-disrupting potential [12,14]. Nevertheless, clinical evidence is doubtful and unlikely to support the proposed link between lavender and tea tree EOs and endocrine disruption in children, due to the actual absence of these ingredients in the offending products [15]. Endocrine-disrupting chemicals (EDCs) are defined by the World Health Organization as exogenous substances or mixtures that alter function(s) of the endocrine system and consequently cause adverse health effects in an intact organism, its progeny or (sub)populations [16]. Published studies dealing with endocrine disruption and EOs or their components have not fully met this definition because the authors have focused on the disruption of steroid hormones, but they have not investigated the potential adverse health effects in connection with the hormonal disruption.

The hPlacentox assay, selected by the public–private PEPPER platform for the pre-validation of methods for endocrine disruptors characterization, is based on the human placental JEG-Tox cell model and allows the study of not only hormones disruption (both steroids and polypeptides), but also adverse health effects in the same cells [17], to meet WHO’s definition of EDCs [18]. Our previous studies in JEG-Tox cells showed that P2X7 receptor activation would be a common cellular mechanism of toxicity for EDCs in placenta [19,20]. P2X7 receptor activation is reported to be involved in multiple pathologies from immune disorders to degenerative diseases and placental disorders [21,22,23,24,25].

The aim of the present study was to evaluate the placental toxicity of some of the most used EOs in the world (orange, niaouli, tea tree, ylang-ylang and wintergreen), and their potential endocrine-disrupting effects. To achieve our goal, we first studied the release of human hyperglycosylated chorionic gonadotropin (h-hCG), human placental lactogen (hPL), estradiol and progesterone, and the activation of P2X7 receptor in human placental JEG-Tox cells after incubation with the whole EOs. To better understand EOs effects, we then studied the same biomarkers after incubation of JEG-Tox cells with EOs’ key components: 1,8-cineol (main component of niaouli EO), limonene (main component of orange EO), 4-terpineol (main component of tea tree EO), benzyl salicylate (suspected endocrine-disrupting component of ylang-ylang EO) and methyl salicylate (main component of wintergreen EO).

## 2. Results

The analysis of EOs revealed that there was 56.34% of 1,8-cineol in niaouli EO, 95.18% of limonene in orange EO, 36.98% of 4-terpineol in tea tree EO, 94.56% of methyl salicylate in wintergreen EO and 2.16% of benzyl salicylate in ylang-ylang EO (Table 1). To better characterize the composition of essential oils, we also studied the potential pesticide contamination. The analysis of more than 250 pesticides by GC–MS/MS method highlights that the tested essential oils did not contain any pesticide (data not shown).

Before studying hormones release and P2X7 receptor activation, we investigated JEG-Tox cell viability after incubation with the EOs. Any concentration inducing a loss of cell viability of at least 30% was considered as cytotoxic [26] and rejected for subsequent analyses dealing with hormones and P2X7 receptor. For clarity’s sake, results for each EO are presented independently from the other EOs. Every EO was tested at three concentrations: 0.17 × 10^−3^%, 0.17 × 10^−2^% and 0.17 × 10^−1^%, according to the literature [12,14].

**Figure 1:**
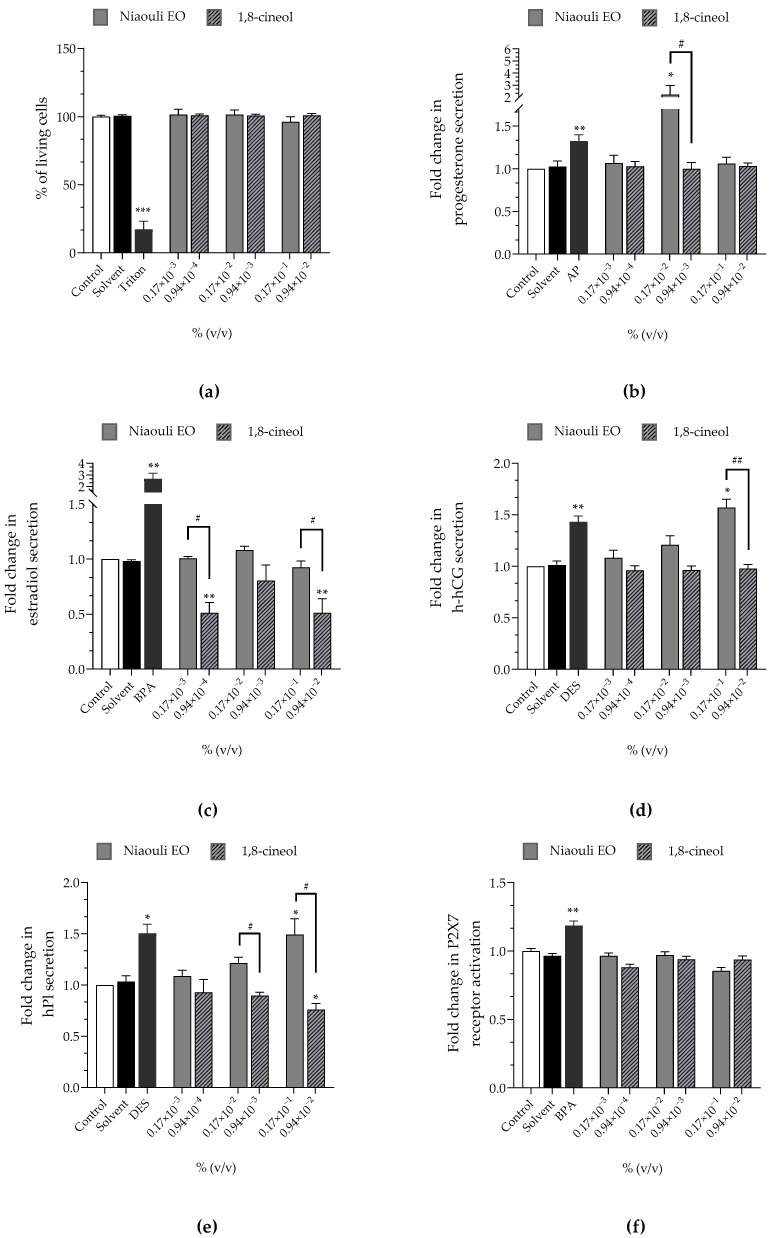
Effects of niaouli EO and 1,8-cineol on (**a**) cell viability (Alamar Blue assay), on released hormones: (**b**) progesterone (**c**) estradiol, (**d**) h-hCG and (**e**) hPL and on (**f**) P2X7 receptor activation (YO-PRO-1 assay) were evaluated, after incubation of JEG-Tox cells for 72 h. Triton^®^ X-100 at 0.016%, bisphenol A (BPA) at 20 µM, 4-tert-amylphenol (AP) at 10 µM and diethylstilbestrol (DES) at 3.75 µM were used as positive controls. The significance thresholds were * *p* < 0.1, ** *p* < 0.01, *** *p* < 0.001, # *p* < 0.1, and ## *p* < 0.01.

Niaouli EO at 0.17 × 10^−3^%, 0.17 × 10^−2^% and 0.17 × 10^−1^% corresponded to 0.94 × 10^−4^%, 0.94 × 10^−3^% and 0.94 × 10^−2^% of 1,8-cineol, respectively, since the tested batch contained 56.34% of 1,8-cineol (Table 2). Niaouli EO and 1,8-cineol induced neither a loss of cell viability nor the P2X7 receptor activation (Figure 1a,f). Niaouli EO at 0.17 × 10^−2^% led to an elevated progesterone secretion (×2.21 compared to the negative control) and an elevated h-hCG secretion at 0.17 × 10^−1^% (×1.57 compared to the negative control), unlike 1,8-cineol (Figure 1b,d). EO induced a higher hPL hormone secretion at 0.17 × 10^−2^% than the control (×1.21) and this increase became significant at 0.17 × 10^−1^%, (×1.49), while 1,8-cineol led to a lower hPL secretion than the control at 0.94 × 10^−3^% and 0.94 × 10^−2^% (×0.89 and ×0.76 at Figure 1e). 1,8-cineol lowered estradiol release at 0.94 × 10^−4^% and 0.94 × 10^−2^% (×0.51), while niaouli EO had no effect on estradiol (Figure 1c).

The rise of hPL secretion induced by niaouli EO was concentration-dependent. Conversely, the variations of the other hormones induced by niaouli EO or 1,8-cineol were not concentration-dependent.

**Figure 2:**
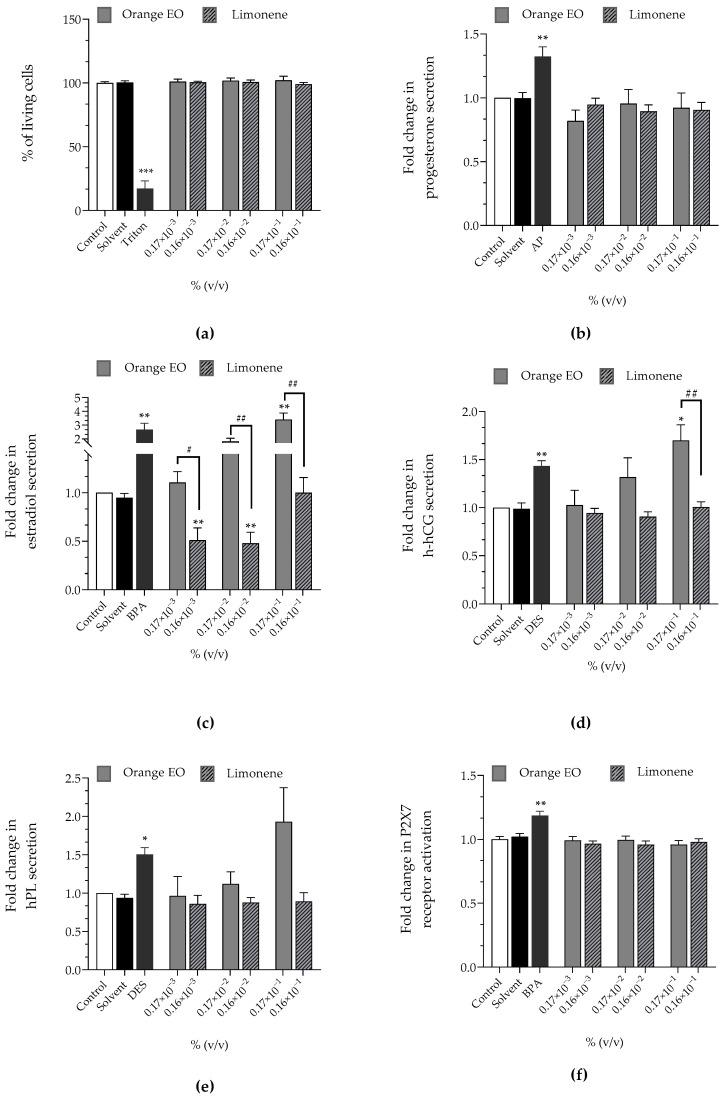
Effects of orange EO and limonene on (**a**) cell viability (Alamar Blue assay), on released hormones: (**b**) progesterone (**c**) estradiol, (**d**) h-hCG, (**e**) hPL and on (**f**) P2X7 receptor activation (YO-PRO-1 assay) were evaluated, after incubation of JEG-Tox cells for 72 h. Triton^®^ X-100 at 0.016%, bisphenol A (BPA) at 20 µM, 4-tert-amylphenol (AP) at 10 µM and diethylstilbestrol (DES) at 3.75 µM are used as positive controls. The significance thresholds were * *p* < 0.1, ** *p* < 0.01, *** *p* < 0.001, # *p* < 0.1, and ## *p* < 0.01.

Orange EO at 0.17 × 10^−3^%, 0.17 × 10^−2^% and 0.17 × 10^−1^% corresponded to 0.16 × 10^−3^%, 0.16 × 10^−2^% and 0.16 × 10^−1^% of limonene, respectively, since the tested batch contained 95.18% limonene (Table 3). Orange EO and limonene did not induce any loss of viability, activation of the P2X7 receptor, or disruption of progesterone secretion (Figure 2a–f). Orange EO significantly raised estradiol secretion to 0.17 × 10^−2^% and 0.17 × 10^−1^% (×1.81 and ×3.41, respectively), while limonene induced a lower secretion than the control at 0.16 × 10^−3^% and 0.16 × 10^−2^% (×0.51 and 0.48, respectively, Figure 2c). Orange EO raised h-hCG secretion to 0.17 × 10^−1^% (×1.70, Figure 2d), but limonene had no effect on h-hCG. Orange EO stimulated hPL secretion at 0.17 × 10^−1^% (×1.93) contrary to limonene (Figure 2e).

The rises of estradiol and h-hCG induced by orange EO were concentration-dependent, while the variations of hPL and estradiol induced by orange EO or limonene were not concentration-dependent.

**Figure 3:**
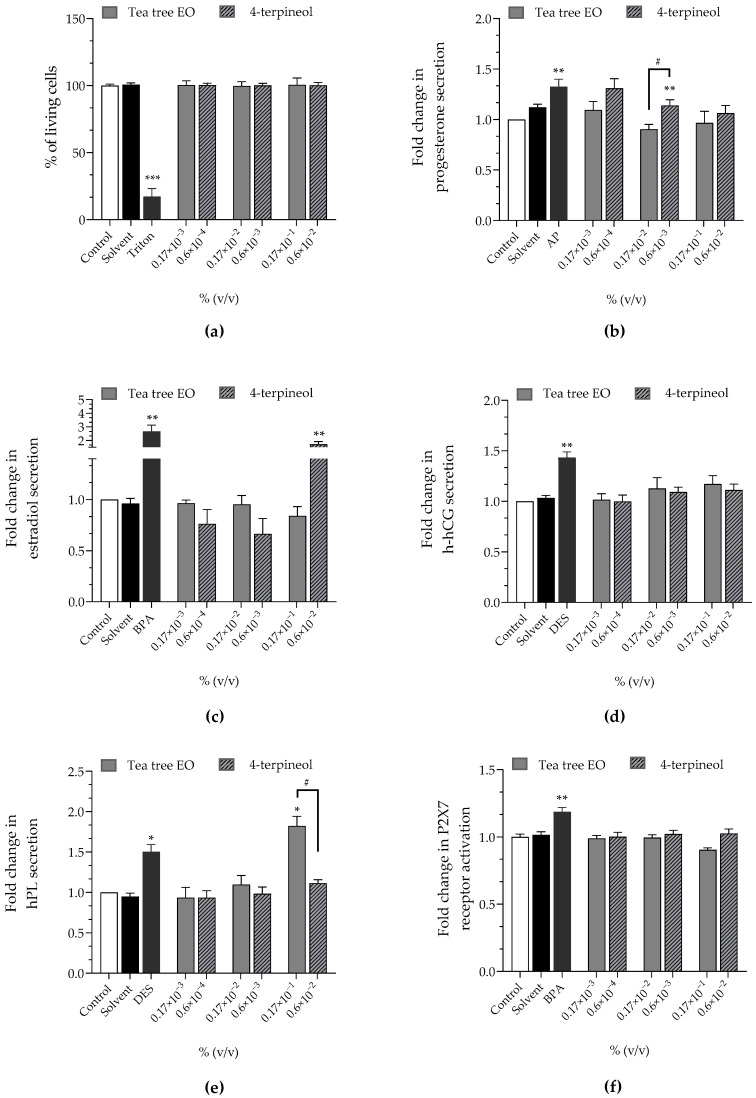
Effects of tea tree EO and 4-terpineol on (**a**) cell viability (Alamar Blue assay), on released hormones: (**b**) progesterone (**c**) estradiol, (**d**) h-hCG, (**e**) hPL and on (**f**) P2X7 receptor activation (YO-PRO-1 assay) were evaluated, after incubation of JEG-Tox cells for 72 h. Triton^®^ X-100 at 0.016%, bisphenol A (BPA) at 20 µM, 4-tert-amylphenol (AP) at 10 µM and diethylstilbestrol (DES) at 3.75 µM are used as positive controls. The significance thresholds were * *p* < 0.1, ** *p* < 0.01, *** *p* < 0.001 and # *p* < 0.1.

Tea tree EO at 0.17 × 10^−3^%, 0.17 × 10^−2^% and 0.17 × 10^−1^% corresponded to 0.6 × 10^−4^%, 0.6 × 10^−3^% and 0.6 × 10^−2^% of 4-terpineol, respectively, since the tested batched contained 36.98% of 4-terpineol (Table 4). Tea tree EO and 4-terpineol did not induce any loss of viability, P2X7 receptor activation or h-hCG disruption. 4-Terpineol induced a higher progesterone secretion than the control at 0.6 × 10^−4^% and 0.6 × 10^−3^% (×1.31 and ×1.14, respectively) and estradiol at 0.6 × 10^−2^% (×1.71), while tea tree EO had no effect on progesterone and estradiol (Figure 3b,c). Conversely, tea tree EO stimulated the secretion of hPL at 0.17 × 10^−1^%. (×1.38) but 4-terpineol did not.

The rises of hormonal secretion induced by tea tree EO and 4-terpineol were not concentration-dependent.

**Figure 4:**
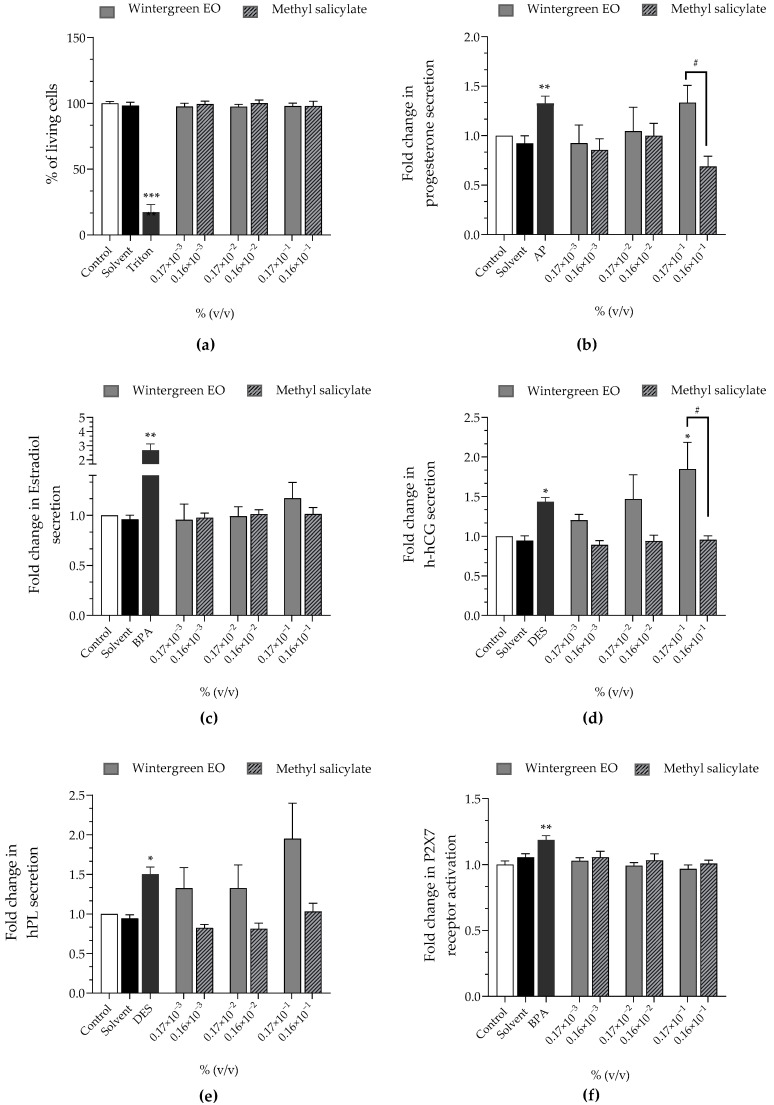
Effects of wintergreen EO and methyl salicylate on (**a**) cell viability (Alamar Blue assay), on released hormones: (**b**) progesterone (**c**) estradiol, (**d**) h-hCG, and (**e**) hPL and on (**f**) P2X7 receptor activation (YO-PRO-1 assay) were evaluated, after incubation of JEG-Tox cells for 72 h. Triton^®^ X-100 at 0.016%, bisphenol A (BPA) at 20 µM, 4-tert-amylphenol (AP) at 10 µM and diethylstilbestrol (DES) at 3.75 µM are used as positive controls. The significance thresholds were * *p* < 0.1, ** *p* < 0.01, *** *p* < 0.001 and # *p* < 0.1.

Wintergreen EO at 0.17 × 10^−3^%, 0.17 × 10^−2^% and 0.17 × 10^−1^% corresponded to 0.16 × 10^−3^%, 0.16 × 10^−2^% and 0.16 × 10^−1^% of methyl salicylate, respectively, since the tested batch contained 94.56% of methyl salicylate (Table 5). Both wintergreen EO and methyl salicylate did not induce any loss of cell viability or activation of the P2X7 receptor at the tested concentrations (Figure 4a,f). Wintergreen EO significantly stimulated progesterone secretion at 0.17 × 10^−1^% (×1.33 compared to the control) contrary to methyl salicylate that reduced progesterone secretion at 0.16 × 10^−1^% (×0.69). EO also stimulated h-hCG secretion at 0.17 × 10^−2^% (×1.47) and this stimulation was significant at 0.17 × 10^−1^% (×1.85), while methyl salicylate had no effect on h-hCG (Figure 4d). Wintergreen EO stimulated hPL secretion (×1.32 at 0.17 × 10^−3^%, and 0.17 × 10^−2^%; ×1.95 at 0.17 × 10^−1^%), but not methyl salicylate, which had no effect on hpL secretion (Figure 4e). Wintergreen EO and methyl salicylate had no effect on estradiol secretion in JEG-Tox cells (Figure 4d).

All hormonal alterations induced by wintergreen EO and methyl salicylate were concentration-independent, except for the rise of h-hCG secretion induced by wintergreen EO.

**Figure 5:**
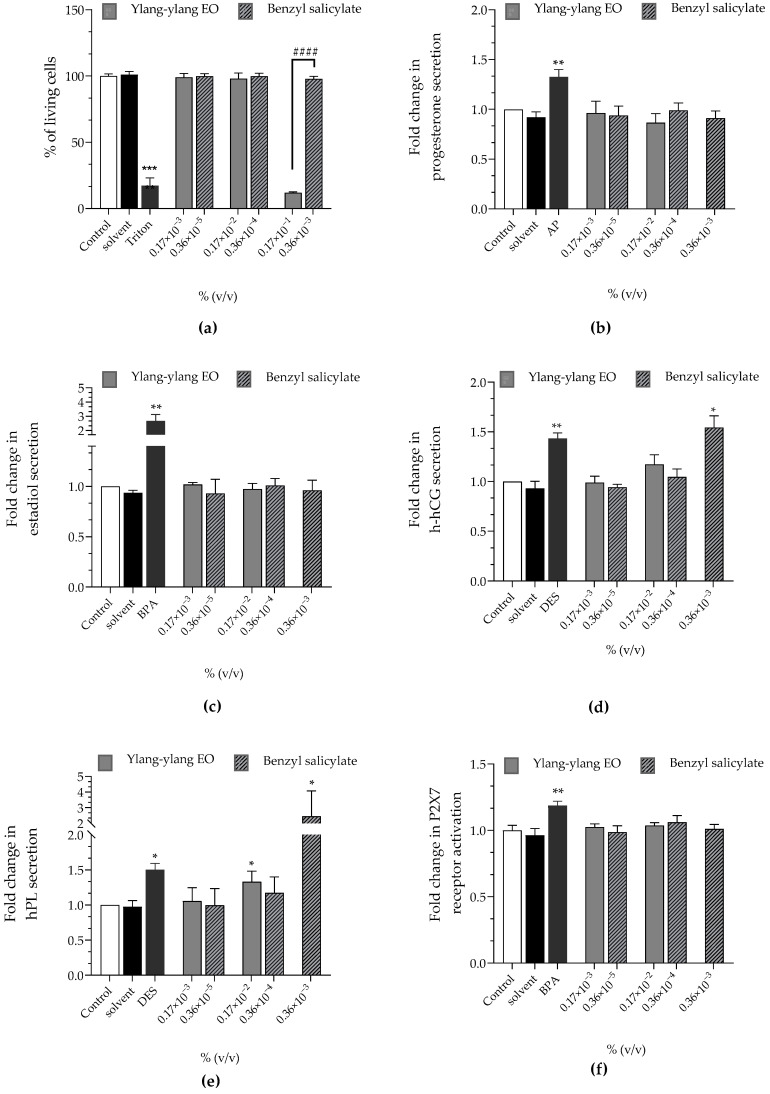
Effects of ylang-ylang EO and benzyl salicylate on (**a**) cell viability (Alamar Blue assay), on released hormones: (**b**) progesterone (**c**) estradiol, (**d**) h-hCG, and (**e**) hPL and on (**f**) P2X7 receptor activation (YO-PRO-1 assay) were evaluated, after incubation of JEG-Tox cells for 72 h. Triton^®^ X-100 at 0.016%, bisphenol A (BPA) at 20 µM, 4-tert-amylphenol (AP) at 10 µM and diethylstilbestrol (DES) at 3.75 µM are used as positive controls. The significance thresholds were * *p* < 0.1, ** *p* < 0.01, *** *p* < 0.001 and #### *p* < 0.0001.

Ylang-ylang EO at 0.17 × 10^−3^%, 0.17 × 10^−2^% and 0.17 × 10^−1^% corresponded to 0.36 × 10^−5^%, 0.36 × 10^−4^% and 0.36 × 10^−3^% of benzyl salicylate, respectively, since the tested batch contained 2.16% of benzyl salicylate (Table 6). Ylang-ylang EO caused a loss of viability at 0.17 × 10^−1^% (12% of living cells) but benzyl salicylate did not (Figure 5a). Cytotoxic concentrations were excluded from subsequent assays. Ylang-ylang EO and benzyl salicylate did not activate the P2X7 receptor (Figure 5f) and had no effect on progesterone nor estradiol secretion (Figure 5b,c). Ylang-ylang EO significantly upregulated hPL secretion at 0.17 × 10^−2^% (×1.33 compared to the control). At the same concentration, benzyl salicylate had no effect but it significantly enhanced hPL secretion at 0.36 × 10^−3^% (×2.45 compared to the control, Figure 5e). Benzyl salicylate significantly raised the secretion of h-hCG to 0.36 × 10^−3^% (×1.54 compared to the control) while ylang-ylang EO had no effect on this hormone (Figure 5d).

All hormonal alterations induced by ylang-ylang EO and benzyl salicylate were concentration-independent.

## 3. Discussion

The aim of this study was to explore and compare the potential endocrine-disrupting effects of five of the most used EOs in the world: tea tree, niaouli, orange, wintergreen and ylang-ylang EOs, and their key compounds: 4-terpineol, 1,8-cineol, limonene, methyl salicylate and benzyl salicylate, in placental cells. The tested concentrations were in accordance with the literature and normal use of EOs [12,14].

Placenta is a target organ for toxic agents including EDCs [27,28,29], where they can be concentrated [4]. During pregnancy, women are particularly vulnerable to EDCs because prolonged or repeated hormonal disruption during pregnancy can lead to severe pathologies for both the mother and the fetus [30,31,32]. We chose to study hormones that play crucial roles during pregnancy and whose dysregulations may lead to severe pregnancy disorders. Altered levels of h-hCG are characteristics of pregnancy complications leading to preeclampsia [33] and placenta accreta [34], the two major causes of severe maternal morbidity and mortality [35]. Progesterone, estradiol and hPL levels are significantly altered in pregnant women who develop gestational diabetes mellitus [36,37], which is a risk factor for perinatal complications such as preterm birth [38] and diabetic mothers [36]. The disruption of maternal estradiol and hCG may also have consequences for fetal and infant development, especially in brain maturation [39].

All these hormone-associated disorders share a common cellular biomarker: the P2X7 receptor activation. Indeed, the P2X7 receptor activation is induced in the above cited, including disorders preterm birth and preeclampsia [25,40], central nervous system diseases [41], and diabetes [42]. Our previous studies showed that the P2X7 receptor activation would be a common cellular mechanism of toxicity for EDCs in placenta [19,20].

For this reason, we selected the hPlacentox assay, based on the human placental JEG-Tox cell model to study the release of hormones in cell supernatants and the activation of P2X7 receptor after incubation with tea tree, niaouli, orange, wintergreen and ylang-ylang EOs, and their key compounds.

This study is the first to explore placental toxicity of EOs. On the one hand, niaouli, orange, tea tree, wintergreen and ylang-ylang EOs disrupted at least one of the studied hormones. On the other hand, we found that none of the tested EOs activated the P2X7 receptor. We are tempted to conclude that EOs seem to be hormone modulators rather than endocrine disruptors since they altered hormones but did not cause adverse cellular effects in our study. The results we obtained with tea tree EO (no alteration of estradiol release) appear in contradiction with previous in vitro studies that demonstrated estrogenic effects of tea tree EO in MCF-7 human breast cells [12,14]. The difference between these results and ours may be explained by the difference in tea tree EOs quality. We purposely selected pesticide-free EOs for our study, whereas no information on pesticide content of the tea tree EO used in the previous study was available. Pesticides can have endocrine-disrupting properties [43,44,45]; chlorpyrifos [46], one of the most frequently found in EOs [47,48], has estrogenic effect [49,50]. It is therefore of the highest importance to better characterize EOs before publishing results.

To better understand the effects of the tested EOs on hormone release, we tested their key compounds in the same conditions. 1,8-cineole, limonene, 4-terpineol, methyl salicylate and benzyl salicylate did not disrupt hormone release in the same way as their corresponding EOs. 1,8-cineol, which represents about 55% of niaouli EO, induced lower secretions of estradiol and hPL than control cells whereas niaouli EO raised them. 4-terpineol, composing about 37% of tea tree EO, had progestational and estrogenic effects that the tea tree EO did not have, but it did not raise hPL, unlike tea tree EO. Benzyl salicylate, approximately present at 2% in ylang-ylang EO, induced higher hPL and h-hCG secretions than control cells whereas ylang-ylang EO only altered hPL secretion. Limonene and methyl salicylate constitute about 95% of orange and wintergreen EO, respectively. Limonene induced an antiestrogenic effect, contrary to orange EO that induced an estrogenic effect; methyl salicylate induced an antiprogestational effect, opposite to wintergreen EO, which induced a progestational effect. Our results showed that none of the five compounds activated P2X7 receptor, like the tested EOs, leading to the same conclusion: these compounds seem to be hormonal modulators rather than endocrine disruptors. It is interesting to note that the most abundant compounds (95% of the total EO) did not have the same effects as the whole oil. This means that the other compounds, even very minor, also participate in the hormonal effects of EOs. Two hypotheses can be raised. First, the additive or synergistic effects, also called the cocktail effects, of all the components are a parameter to be considered when evaluating EOs, particularly in the field of EDCs. Second, the minor compounds could be responsible for the hormonal effects of EOs. Indeed, unlike other toxic substances, EDCs do not necessarily exert their effects in a dose-dependent manner.

In 2019, benzyl salicylate was added to the list of the 14 substances to be screened as potential endocrine disruptors by the European Scientific Committee for Consumer Safety (SCCS) and was included in the Community rolling action plan (CoRAP) of the European REACH regulation to be assessed for endocrine disruption. Indeed, in vitro and in vivo published studies have showed that benzyl salicylate alters estrogens. The yeast estrogen screen (recombinant yeast expressing estrogen receptor α) and the E-screen assay (proliferation of MCF-7 cells), in vitro assays showed estrogenic, antiestrogenic or even no effects of benzyl salicylate [51,52,53,54,55,56]. The only reported in vivo evidence of an estrogenic effect induced by benzyl salicylate, although very weak, is the study by Zhang et al. using the uterotrophic assay in mice and rats [57]. Regarding 1,8-cineol, limonene and 4-terpineol, one in vitro study in the human breast cancer MCF-7 cell line showed an estrogenic effect of 4-terpineol, but not of limonene nor 1,8-cineol [14]. We did not demonstrate any change in estradiol release with 1,8-cineol, limonene, 4-terpineol and benzyl salicylate in human placental cells. Discrepancies may be directly related to the differences in the models. The recombinant yeast assay uses a unicellular eukaryotic organism as a cell model, which is physiologically and genetically far from the human organism. It is therefore difficult to extrapolate data obtained from yeasts to humans. Human breast MCF-7 cells, deriving from the human mammary gland, possess the receptors to respond to estrogens or estrogenic molecules but are not able to secrete hormones. Indeed, the mammary gland is an exocrine and not an endocrine gland, whereas the placenta is an endocrine organ able to produce, secrete and respond to several hormones such as estradiol. Studies performed in human breast MCF-7 cells are therefore focused either on their proliferation under the influence of estrogenic molecules, or on the transcriptional activity of estrogen receptor α [14], but not on estradiol release. Yet, transcriptional activation does not necessarily lead to physiological consequences; cellular posttranscriptional regulatory mechanisms can counterbalance transcription activation through translation repression [58]. The in vivo uterotrophic assay in rodents measures estrogenic activity by studying the variation in uterine weight. The uterus is not an endocrine organ; it can respond to hormones but cannot secrete any of them. All these observations lead to the hypothesis that EDCs act differently depending on the tissue (uterus, mammary gland and placenta), which is in accordance to our previous results obtained in lung, skin and placental cells [20].

Our study highlights the difficulty and complexity of predicting the endocrine disrupting potential of EOs, based only on the safety data related to one of their components, even a major one. Each EO contains many different chemicals that vary in structure and composition, some EOs contain over 150 substances. It is therefore essential to study the entire EO rather than its individual components in order to conclude on a potential effect of EOs. Furthermore, according to our results, tea tree, niaouli, orange, wintergreen and ylang-ylang EOs appear to be more hormonal modulators than endocrine disruptors in placenta, as they did not activate P2X7 receptor. To confirm this statement, it would be necessary to perform further in vitro experiments to study other adverse health effects at the cell level. Mitochondrial alterations, already identified as key elements in understanding placental disorders induced by EDCs [20], could be, for example, assessed. This study also underlines the need to differentiate EDCs from hormonal modulators, which are certainly less dangerous. Many components of EO are also present in everyday products such as food and beverages that are important for pregnant women’s health, and they do not necessarily provoke endocrine disruption. Indeed, the human body has an endocrine feedback system to limit hormonal imbalance [59,60]. We can hypothesize that hormonal modulations are most of the time counterbalanced to avoid pathological consequences but must serve as a warning signal.

## 4. Materials and Methods

Chemicals and reagents. Minimum essential medium (MEM), fetal bovine serum (FBS), 2 mM glutamine, 10,000 U/mL penicillin and 10,000 µg/mL streptomycin, trypsin-EDTA 0.05% and phosphate buffer saline (PBS) were provided by Gibco (Paisley, UK) and cell culture plastics such as flasks and microplates by Corning (Schiphol-Rijk, The Netherlands). YO-PRO-1^®^ was obtained from ThermoFisher Scientific (Waltham, Massachusetts, USA), and Alamar Blue probes from Alfa aesar (Haverhill, Massachusetts, USA). Hyperglycosylated hCG and hPL ELISA kit were purchased from MyBioSource (San Diego, California, USA) and estradiol and progesterone from Cisbio (Codolet, France). The niaouli EO (*Melaleuka (M.) viridiflora*, which is a synonym for *M. quinquenervi*), orange EO (*Citrus sinensis*), tea tree EO (*M. alternifolia*), wintergreen EO (*M. alternifolia*), the ylang-ylang EO (*Cananga odorata*) were obtained from Laboratoires Léa nature (Périgny, France). EOs components, benzyl salicylate (catalog #84260), methyl salicylate (catalog #M6752), 4-terpineol (catalog #03900590), limonene (catalog #8.14546) and 1,8-cineol (catalog #C80601) were purchased from Sigma-Aldrich (Saint Quentin Fallavier, France). Positive controls were used to ensure that the cell model responded correctly in our experimental conditions and were obtained from Sigma-Aldrich.

EOs’ composition analysis: the composition of all tested EOs was analyzed by GC–MS analysis according to NF ISO 11024 standard with the SHIMADZU GC-FID 2010 PLUS/MS QP 2010SE, column RxiR 5 SILM 60 m, 0.25 mm ID and 0.25 µM df.

The following analytical conditions were used for both apparatus and columns: the oven temperature was programmed at 50 °C, held for 5 min, then increased to 240 °C at a rate of 3 °C/min; the injector temperature was set at 240 °C; the carrier gas was helium with a flow rate of 1 mL/min; a splitting ratio of 1:50 was applied; the injection volume was set to 1 μL. For the GC–MS mass spectrometry interface, the MS source temperature was set at 220 °C with an ionization energy of 70 eV and an interface temperature of 240 °C. A full scan was recorded (50–700 *m*/*z*).

The identification of constituents was achieved by comparing mass spectra with the Mass Spectra Library (NIST 98) compounds and by comparison of the retention indices (RI) calculated from the injection of a C8–C20 hydrocarbons alkanes mixture with retention indices from the literature on both columns. Some selected pure standards were also used.

Human placental cell culture: The JEG-3 human trophoblast cell line was obtained from the American Type Culture Collection (ATCC HTB-36). Cells were cultured in minimum essential medium (MEM) supplemented with 10% fetal bovine serum (FBS), 1% L-glutamine, 0.5% penicillin and streptomycin, in 75 cm^2^ polystyrene flasks. Cell cultures were maintained in a cell culture incubator (37 °C, saturated humidity, 5% CO_2_). When the JEG-3 cells reached subconfluency, they were detached using trypsin-EDTA and counted. The cellular suspension was diluted and seeded in 96-well microplates at a cellular density of 80,000 cells/mL (200 µL/well), then kept at 37 °C for 24 h. Stock solutions of niaouli, orange, tea tree, wintergreen and ylang-ylang EOs were obtained after dilution to 2/3 in absolute ethanol, and then diluted in MEM supplemented with 2.5% FBS to obtain three concentrations: 0.17 × 10^−3^%, 0.17 × 10^−2^% and 0.17 × 10^−1^% (*v*/*v*). Cells were incubated for 72 h with the different concentrations of EOs, according to the literature [12,14], in MEM supplemented with 2.5% FBS according to Olivier et al.’s protocol that describes the JEG-Tox model [18]. EOs’ components were diluted under the same conditions as EOs and tested at similar percentages as those found in the EOs. The final concentration of ethanol on cells was less than or equal to 0.008%.

Cell viability—Alamar Blue assay: The Alamar Blue stock solution (0.1 mg/mL) was prepared in PBS buffer and stored at 4 °C away from light. The working solution used for the test was obtained by diluting the stock solution at 1/11 in culture medium supplemented with 2.5% FBS. After removing the supernatants and rinsing the cells with PBS, the Alamar Blue working solution was distributed to the wells. Then, the microplate was placed in the incubator for 6 h and read (λ_ex_ = 535 nm and λ_em_ = 600 nm) with a Tecan Spark^®^ microplate reader (Männedorf, Switzerland). Triton^®^ X-100 was used as a positive control for cytotoxicity in the Alamar Blue assay.

Placental and sexual hormones quantification: After centrifugation of the 96-well microplates, the cell supernatants were collected and the hormones were quantified: human hyperglycosylated chorionic gonadotropin (h-hCG), human placental lactogen (hPL) by ELISA according to the supplier’s instructions (MyBioSource), and estradiol and progesterone by FRET according to the supplier’s instructions (Cisbio). The Spark^®^ microplate reader was used for both techniques. Substances of very high concern (SVHC) due to their endocrine-disrupting properties were used as positive controls: bisphenol A (BPA) for estradiol release, 4-tert-amylphenol (AP) for progesterone release and diethylstilbestrol for h-hCG and hPL releases.

Cell death P2X7 receptor activation—YO-PRO-1^®^ assay: P2X7 cell death receptor activation was evaluated using the YO-PRO-1^®^ assay [61]. The YO-PRO-1^®^ probe only enters into cells after pore opening induced by P2X7 receptor activation and binds to DNA, emitting fluorescence. A 1 mM YO-PRO-1 stock solution was diluted at 1/500 in PBS just before use and distributed in the wells of the microplate. After a 10 min incubation time at room temperature, the fluorescence signal was read (λ_ex_ = 485 nm, λ_em_ = 531 nm) using a Spark^®^ microplate reader. BPA was used as a positive control for P2X7 receptor activation in JEG-Tox cells [19,20].

Results exploitation and statistical analysis: Results are expressed in percentage or fold change compared to control cells and presented as means of at least three independent experiments ± standard errors of the mean. A statistical analysis was performed using GraphPad Prism 8 software (La Jolla, CA, USA). A one-way analysis of variance followed by a Dunnett’s test with the risk α set at 5% was performed to compare the EOs’ and components’ incubation with a negative control (*p*-values expressed with the symbol *), and to compare EOs with their components at the same concentration (*p*-value expressed with the symbol #). The significance thresholds were * *p* < 0.1, ** *p* < 0.01, *** *p* < 0.001, # *p* < 0.1, ## *p* < 0.01 and #### *p* < 0.0001.

## 5. Conclusions

Different points have been addressed in this paper. First, we demonstrated that niaouli, orange, tea tree, wintergreen and ylang-ylang EOs appear to be hormonal modulators rather than endocrine disruptors in human placental cells. Second, the key components of EOs did not have the same hormonal effects as whole EOs, proving the complexity of EOs and the need to fully characterize EOs in terms of components and pesticides before launching any safety assessment. In order to conclude on the potential toxic effects of EOs on the placenta, it is thus essential to study whole EOs rather than their components individually.

## Figures and Tables

**Table 1:** Composition analysis of the tested essential oil batches.

EO	Component	Relative Proportion (%)
niaouli	1–8 cineol	56.34
orange	limonene	95.18
tea tree	4-terpineol	36.98
wintergreen	methyl salicylate	94.56
ylang-ylang	benzyl salicylate	2.16

**Table 2:** Summary of the results of niaouli EO compared to 1,8-cineole.

	Niaouli EO	1,8-cineol
Cell viability	approx. 100%	approx. 100%
Progesterone	↗ (×2.21 at 0.17 × 10^−2^%)	approx. ×1
Estradiol	approx. ×1	↘ (×0.51 at 0.94 × 10^−4^% and 0.94 × 10^−2^%)
h-hCG	↗ (×1.57 at 0.17 × 10^−1^%)	approx. ×1
hPL	↗ (×1.21 at 0.17 × 10^−2^%)↗ (×1.49 at 0.17 × 10^−1^%)	↘ (×0.89 at 0.94 × 10^−3^%)↘ (×0.76 at 0.94 × 10^−2^%)
P2X7 receptor activation	approx. ×1	approx. ×1

**Table 3:** Summary of the results of orange EO compared to limonene.

	Orange EO	Limonene
Cell viability	approx. 100%	approx. 100%
Progesterone	approx. ×1	approx. ×1
Estradiol	↗ (×1.81 at 0.17 × 10^−^^2^%)↗ (×3.41 at 0.17 × 10^−^^1^%)	↘ (×0.51 at 0.16 × 10^−3^%)↘ (×0.48 at 0.16 × 10^−2^%)
h-hCG	↗ (×1.70 at 0.17 × 10^−1^%)	approx. ×1
hPL	↗ (×1.93 at 0.17 × 10^−1^%)	approx. ×1
P2X7 receptor activation	approx. ×1	approx. ×1

**Table 4:** Summary of the results of tea tree EO compared to 4-terpineol.

	Tea Tree EO	4-Terpineol
Cell viability	approx. 100%	approx. 100%
Progesterone	approx. ×1	↗ (×1.31 at 0.6 × 10^−4^%)↗ (×1.14 at 0.6 × 10^−3^%)
Estradiol	approx. ×1	↗ (×1.71 at 0.6 × 10^−2^%)
h-hCG	approx. ×1	approx. ×1
hPL	↗ (×1.38 at 0.17 × 10^−1^%)	approx. ×1
P2X7 receptor activation	approx. ×1	approx. ×1

**Table 5:** Summary of the results of wintergreen EO compared to methyl salicylate.

	Wintergreen EO	Methyl Salicylate
Cell viability	approx. 100%	approx. 100%
Progesterone	↗ (×1.33 at 0.17 × 10^−1^%)	↘ (×0.69 at 0.16 × 10^−1^%)
Estradiol	approx. ×1	approx. ×1
h-hCG	↗ (×1.47 at 0.17 × 10^−2^%)↗ (×1.85 at 0.17 × 10^−1^%)	approx. ×1
hPL	↗ (×1.32 at 0.17 × 10^−3^%)↗ (×1.32 at 0.17 × 10^−2^%)↗ (×1.95 at 0.17 × 10^−1^%)	approx. ×1
P2X7 receptor activation	approx. ×1	approx. ×1

**Table 6:** Summary of the results of ylang-ylang EO compared to benzyl salicylate.

	Ylang-ylang EO	Benzyl Salicylate
Cell viability	approx. 100%	approx. 100%
Progesterone	approx. ×1	approx. ×1
Estradiol	approx. ×1	approx. ×1
h-hCG	approx. ×1	↗ (×1.54 at 0.36 × 10^−3^%)
hPL	↗ (×1.33 at 0.17 × 10^−2^%)	↗ (×2.45 at 0.36 × 10^−3^%)
P2X7 receptoractivation	approx. ×1	approx. ×1

## Data Availability

The data presented in this study are available on request from the corresponding author.
